# Axillary artery pseudoaneurysm with concurrent distal thrombosis: a case report

**DOI:** 10.1093/jscr/rjae061

**Published:** 2024-02-13

**Authors:** Runze Wei, Zhaolei Chen

**Affiliations:** Department of Vascular Surgery, Northern Jiangsu People's Hospital, Yangzhou 225001, China; Department of Vascular Surgery, Northern Jiangsu People's Hospital, Yangzhou 225001, China

**Keywords:** axillary artery, pseudoaneurysm, limb ischemia, endovascular therapy

## Abstract

This report details a case of axillary artery pseudoaneurysm with concurrent distal thrombosis, manifesting as acute upper extremity ischemia. The condition was successfully treated with a hybrid surgical approach, employing a covered stent graft and Fogarty balloon thrombectomy. We review the relevant literature on the management of this rare but critical vascular condition.

## Introduction

Pseudoaneurysms in the axillary artery represent a rare clinical phenomenon, with the co-occurrence of distal arterial embolization being an even more infrequent complication. Although endovascular techniques have been established as a standard approach for managing common arterial diseases, literature documenting their application in cases of pseudoaneurysms accompanied by synchronous arterial emboli is limited. This report delineates a case involving acute upper extremity ischemia resulting from an axillary artery pseudoaneurysm with concurrent distal thrombosis, which was successfully managed through hybrid surgery.

## Case report

**Figure 1:**
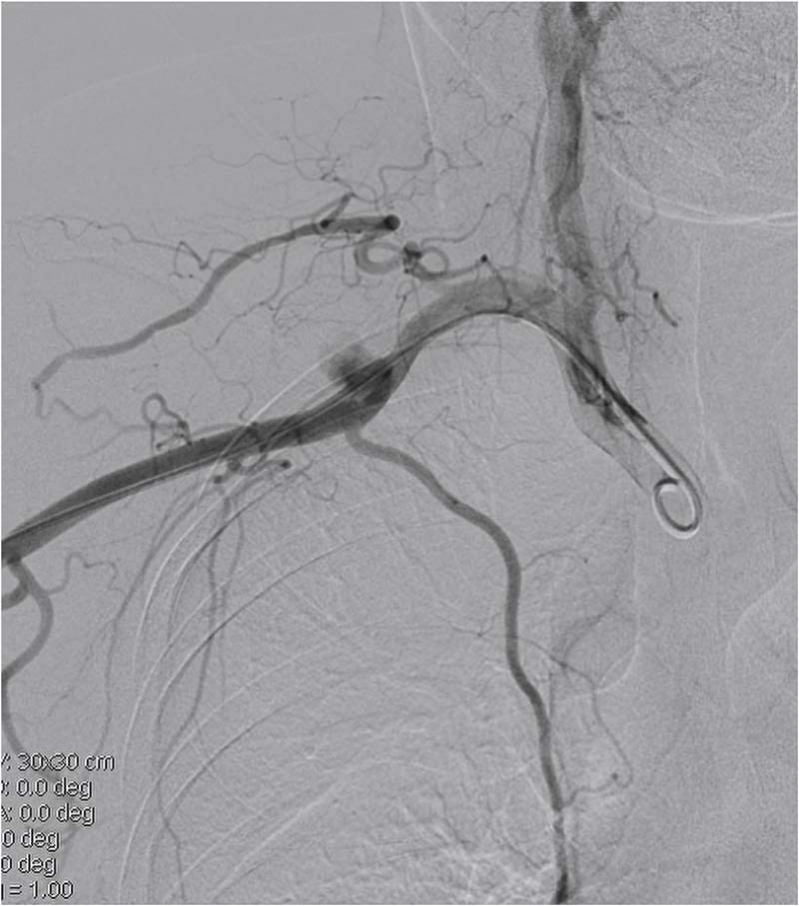
DSA indicates an axillary artery pseudoaneurysm.

**Figure 2:**
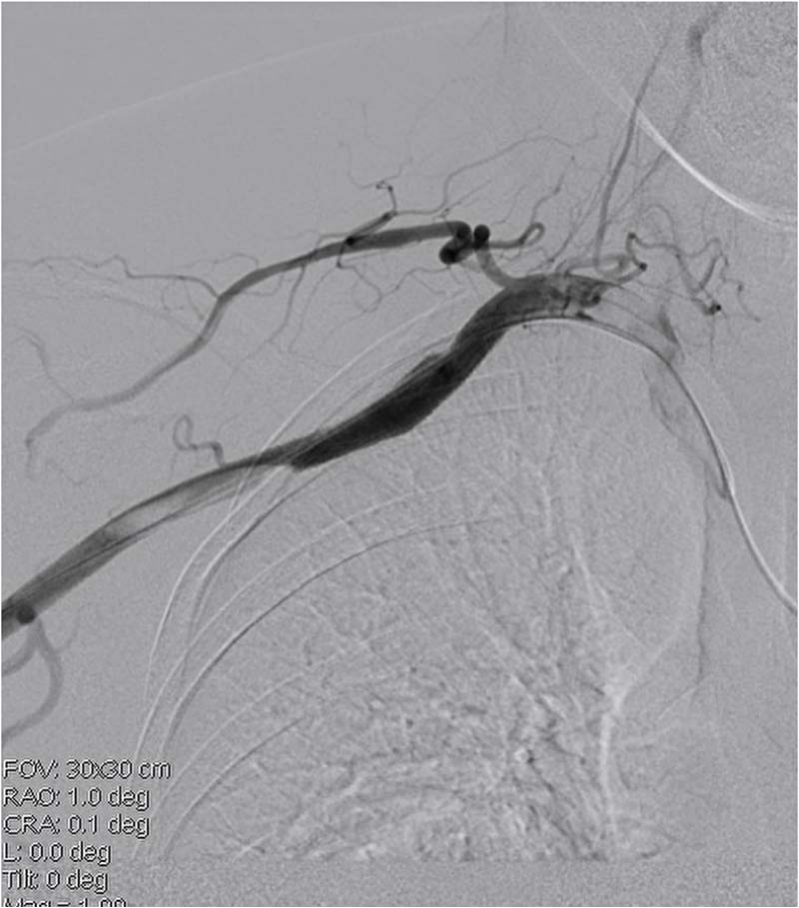
Covered stent placement isolates the pseudoaneurysm rupture, and further thrombectomy treatment is planned.

A 45-year-old female patient presented with a 20-day history of acute onset cooling, pallor, and paresthesia in the right upper limb along with supraclavicular discomfort on the right side. Notably, the patient’s medical history was devoid of significant conditions such as atrial fibrillation, hypertension, or coronary artery disease. Physical examination revealed a marked decrease in skin temperature, the absence of palpable radial and brachial pulses, and pallor in the affected extremity, with no detectable neurological deficits. Vascular ultrasonography revealed arterial thrombosis extending from the subclavian artery to the distal brachial, radial, and ulnar arteries. Computed tomographic angiography further characterized these findings, identifying filling defects in the right axillosubclavian vasculature indicative of a substantial clot burden. Intraprocedural digital subtraction angiography (DSA), conducted to facilitate endovascular thrombectomy, revealed a 12 × 12 mm pseudoaneurysm originating from the axillary artery (Fig. 1). The definitive treatment involved the deployment of an 8 × 60 mm (Fluency, Bard, USA) covered stent graft across the lesion in the vessel, followed by Fogarty balloon thrombectomy to re-establish distal perfusion (Fig. 2). Postoperatively, the patient received aspirin and short-term anticoagulation. Follow-up at 6 months, including clinical and ultrasonographic evaluations, confirmed symptom improvement, pulse restoration, and absence of ischemic or stent-related complications.

## Discussion

Pseudoaneurysms represent a pathological condition characterized by disruptions in the arterial wall’s structural integrity [1]. This leads to blood extravasation into surrounding tissues, followed by encapsulation, forming a pulsatile mass contiguous with the vascular lumen. Congenital pseudoaneurysms are often attributed to aberrant vascular development *in utero*, as seen in connective tissue disorders like Marfan syndrome, or may arise from hereditary predispositions. By contrast, acquired pseudoaneurysms can result from various factors, including atherosclerosis, hypertension, diabetes, trauma, iatrogenic vascular injuries, autoinflammatory vasculitides, inadvertent anticoagulation, intravenous substance abuse, among others.

Clinically, peripheral pseudoaneurysms typically present as a pulsating mass, often accompanied by a thrill or bruit upon auscultation. Expanding pseudoaneurysms may cause symptoms due to mass effect on adjacent structures, such as venous obstruction, limb swelling, sensorimotor deficits from nerve compression, muscular necrosis, or even distal thrombosis, leading to acute limb ischemia. Secondary infection may manifest as erythema, warmth, swelling, and pain. For diagnosis, Doppler ultrasonography serves as a rapid, accessible, and highly accurate modality [2]. Advanced cross-sectional imaging, including CT angiography or MR angiography, provides detailed anatomical information, crucial for treatment planning. DSA, considered as the gold standard, is particularly valuable when pseudoaneurysm morphology or hemodynamics are unclear on noninvasive imaging or if endovascular therapy is under consideration.

Upon diagnosis, pseudoaneurysms necessitate prompt intervention to prevent complications like rupture or distal thromboembolization. Surgical options vary based on anatomical considerations and include focal aneurysmectomy with direct arterial reconstruction, aneurysmectomy with arterial ligation or primary repair, aneurysmectomy with arterial resection and end-to-end anastomosis, and aneurysmectomy with autologous or synthetic interposition grafting [3]. Endovascular techniques, such as covered stent deployment, coil embolization, or direct thrombin injection, offer less-invasive alternatives [4, 5]. The choice between open repair and endovascular intervention depends on the lesion severity, luminal patency, and patient comorbidities.

In summary, axillary artery pseudoaneurysms with distal embolization are rare and complex. Diagnosis involves advanced imaging techniques, and treatment ranges from surgical reconstruction to minimally invasive endovascular procedures. The choice of therapy depends on the lesion’s characteristics and patient factors.
